# Food Security–Renewable Energy Nexus: Innovations and Shocks in Saudi Arabia

**DOI:** 10.3390/foods14101797

**Published:** 2025-05-18

**Authors:** Nourah A. Althani, Raga M. Elzaki, Fahad Alzahrani

**Affiliations:** Department of Agribusiness and Consumer Science, College of Agriculture and Food Sciences, King Faisal University, Al-Ahsa 31982, Saudi Arabia; 223000415@student.kfu.edu.sa (N.A.A.); falzahrani@kfu.edu.sa (F.A.)

**Keywords:** food availability, food stability, solar energy, wind energy, sustainability

## Abstract

The rising global demand for food and energy has led to growing attention to the nexus between food security and renewable energy. This study aims to investigate the impacts and shocks of renewable energy consumption, particularly solar and wind energy, on food availability and stability in Saudi Arabia, by assessing both short-term and long-term effects. We use the time series annual data covering the period (2000–2022) analyzed by applying the Vector Autoregressive (VAR) model system and its environment, Granger causality, the forecast-error variance decompositions (FEVD), and the impulse response functions (IRFs). The VAR results indicated that wind renewable energy positively affects food availability; one unit of wind energy consumption will significantly increase food availability by 3.16% (Z value 2.017 at a 5% significance level), and no statistically significant coefficients are associated with food stability. Also, the results confirmed that one unit of renewable energy consumption from solar will significantly increase food stability by 36.5% in Saudi Arabia (Z-value 1.682 at a 10% significance level). The Granger causality results concluded that solar energy has a bidirectional Granger causality with food availability but not food stability. The FEVD results showed that solar energy shocks have more persistent impacts in explaining the rapid increase in food security than wind energy shocks in both the short and long term. The IRFs concluded that food availability has shown a positive and steady increase in response to wind energy. This study provides practical recommendations for policymakers to balance energy transition goals with food security concerns. Future research should explore emerging technologies in wind and solar energy that can enhance efficiency and sustainability while minimizing adverse effects on food security.

## 1. Introduction

The rising global population, urban migration, economic advancement, technological innovation, and climate change are the primary drivers of increasing pressure on limited and non-renewable resources [1,2,3]. Addressing the challenge of securing sustainable food sources for an expanding global population is a crucial issue that demands attention [4]. Sustainable food security is the solution to improving living standards, but it is impossible without access to modern energy (clean energy). This necessitated the need to resort to renewable energy sources. Therefore, food security and its relationship with renewable energy are the basis for the sustainable production of sufficient food to meet the population’s requirements [5].

Food security and its pillars (i.e., availability, access, utilization, and stability) are disrupted by conflict, governmental instability, or economic crises in both developed and developing countries [6]. Due to increased rates of food supply to achieve food security, the energy sector plays a key role in expanding food production [7]. Moreover, different renewable energy sources may improve food availability, accessibility, utilization, and stability in the long term [8].

Transitioning to modern renewable energy consumption is related to reducing dependence on fossil fuels [9], which can mitigate such pressures by decarbonizing the agricultural supply chain [10,11]. This shift also aligns with the United Nations Sustainable Development Goals (UN SDGs), particularly SDG 7 (Affordable and Clean Energy) and SDG 2 (Zero Hunger) [10]. In addition, the agricultural sector worldwide faces several pressures, including rising food demand due to population growth, constrained access to conventional energy sources, and increasing volatility in energy prices. Therefore, the utilization of renewable energy has been a pressing concern, especially in advanced industrialized countries [12,13]. Enhanced access to renewable energy can directly and indirectly aid in achieving the Sustainable Development Goals [14].

Saudi Arabia is considered one of the leading oil producers in the world, but it realizes the importance of expanding energy sources and investing in renewable and sustainable energy. As renewable energy plays a critical role in enhancing food security [14], Saudi Arabia has directed its efforts to promote the use of renewable energy, as the government announced in its National Transformation Program [15], which aims to expand renewable energy capacities and increase its share in the national energy mix, especially solar and wind energy. Several projects have been implemented to generate electricity from solar energy and construct wind stations, where Saudi Arabia has worked to find innovative solutions represented in the projects of the National Renewable Energy Program, NREP [15], which aims to increase the Kingdom’s share in the production of renewable energy and diversify local energy sources. The International Energy Agency (IEA) indicated that the ratio of total renewable energy production compared to total oil production has reached 0.16%, and the ratio of total natural gas production has reached 0.12% [16].

Solar and wind energy have been identified as key solutions to address energy shortages, while ensuring sustainable agricultural production. Food security and renewable energy are critical sustainable development mechanisms [17], particularly in resource-scarce regions like Saudi Arabia. As the country aims to diversify its economy under Vision 2030, the transition to renewable energy sources such as solar and wind energy is vital in promoting long-term sustainability.

However, the relationship between renewable energy and food security in Saudi Arabia remains complex and underexplored. Currently, most agricultural practices in the country rely heavily on using fossil fuels, increasing GHGs emissions and environmental degradation. Given Saudi Arabia’s unique challenges, including extreme climatic conditions, water scarcity, and dependence on food imports, understanding the renewable energy–food security nexus is critical for evidence-based policymaking [18].

This study is motivated by the potential for integrating renewable energy technologies, such as solar-powered irrigation and wind energy systems, into agricultural production. Such integration could enhance food output, reduce costs, improve food security, and promote sustainability, while mitigating climate change impacts. By simultaneously addressing energy and agricultural needs, Saudi Arabia could develop a more resilient food system and reduce its reliance on imported food.

Therefore, this study aims to examine the effects and shocks of renewable energy consumption, particularly solar and wind energy, on Saudi Arabia’s food security by examining the short-term and long-term effects. The research seeks to provide insights that can guide sustainable energy and agricultural policies in Saudi Arabia by identifying the opportunities and challenges within this nexus.

Concerning the review of empirical studies relevant to this article, recent literature has increasingly applied macroeconomic analyses to topics related to energy and food security [19,20,21,22,23]. At the same time, growing scholarly attention has focused on the opportunities and challenges of integrating solar and wind energy into agricultural systems and their connection with food security [24,25,26,27]. Researchers found that integrating renewable energy into agriculture can enhance food security in several ways, including increasing energy access for smart irrigation and farm management [28], reducing greenhouse gas emissions [25], and stabilizing food production in remote off-grid rural areas [25].

Recent studies examine the modern approach to investigating the sustainability of the supply chain [29,30]. One study has investigated carbon emission reduction in sustainable supply chains using carbon trading mechanisms and employs quantitative modeling under demand uncertainty to evaluate the effectiveness of three contractual types: two-part tariff, revenue sharing, and quantity flexibility. Through comparative analysis, the research assesses each contract’s ability to enhance coordination, increase profitability, and support sustainability goals [31]. Therefore, enhancing supply chain efficiency, reducing emissions, managing market uncertainties, and promoting equitable profit distribution can indirectly contribute to strengthening food security.

A recent study highlights the significant importance of expanding energy storage systems (ESS) as part of renewable energy strategies. It emphasizes that increasing subsidies and promoting investment in infrastructure, particularly for renewable energy and large-scale battery storage, is essential for the successful development and deployment of ESS technologies [9]. Therefore, investing in energy storage systems within renewable energy frameworks not only supports green energy, it also directly strengthens food systems by making agriculture more resilient, efficient, and sustainable. This ultimately contributes to long-term food security.

He et al. [32] applied the Generalized Method of Moments (GMM) approach using panel data and showed that renewable energy has a significant positive impact on food security. Similarly, Çakmakçı et al. [33] observed that renewable energy plays an essential role in supporting food security by providing clean and consistent energy supplies for agricultural performance in developing countries.

A recent study aimed to identify the long-term and short-term associations between renewable energy and food security for South Asian countries, applying the Dynamic Common Correlated (DCC) technique, which showed that the usage of renewable energy sources has a positive consequence on food security in the short run but has no impact on the long run [23]. Furthermore, a study performed in European Union countries employed several econometric analytical techniques, including the Feasible Generalized Least Squares (FGLS) model with Robust Standard Errors (RSE) and a Quantile Regression (QR) model. The results showed that renewable energy consumption has a nonlinear effect on cereal production [34].

A study used a panel smooth transition regression model to investigate the connection between renewable energy consumption and agricultural productivity, demonstrating a positive and significant connection between renewable energy consumption and agricultural productivity [35]. The effects of climate change and energy shock on food were investigated in a study using a panel-VAR model, and the results showed that shock from energy price fluctuations has a positive impact on the price of agricultural commodities [36].

A study conducted across Arab countries employed the Auto-Regressive Distributed Lag (ARDL) model and the Vector Error Correction Model (VECM) to examine the short- and long-term dynamics of the relationship between renewable energy and food security, while also identifying causal linkages among these countries. The ARDL results indicated that renewable energy-driven purification enhances food security [37]. A study conducted in India, designed to investigate the significance of utilizing solar renewable energy to fulfill the energy demands of agricultural activities, revealed that solar power utilization is significant in cost savings, greater energy independence, and minimizing pollution [38].

Recent research has investigated the connection between food security and solar energy, focusing on how solar technologies can enhance agricultural productivity and identify food security challenges [39]. Another study analyzed the impact of solar energy on food processing and preservation and confirmed that solar energy can be utilized in food drying processes to reduce post-harvest losses. It also indicates that solar energy effectively decreases drying times and preserves food quality, making it important for improving food security in agricultural societies [40].

Solar energy presents challenges and trade-offs, such as land use conflicts. The development of ground-based solar energy resources may result in the conversion of farmland, potentially compromising food security. Studies have highlighted that approximately 10% of agricultural land has been repurposed for solar installations [41]. Similarly, recent literature examining the connection between wind renewable energy and food security focuses on how wind energy integration into agricultural systems impacts food production and resource sustainability. Research performed to measure the relationship between energy and food security using Cobb–Douglas production functions combined with advanced econometrics models confirmed that boosting energy security leads to greater food security and indicated that energy is essential to attaining food security and zero hunger in Africa [42].

We conclude that the reviewed literature illustrates that most studies have concentrated on only one type of renewable energy source or renewable energy in general. In this study, we consider two sources, solar and wind. Therefore, the current study seeks to expand on the previous studies by answering the question: Do solar and wind renewable energy significantly affect food security? The answer to this question is important for decision-makers and policy planners, specifically for Saudi Arabia and generally worldwide, to design the full picture of a comprehensive framework for sustainable development with an enhancement in food security.

This study makes several significant contributions to the existing literature on the relationship between food security and renewable energy. It provides empirical evidence from a country that heavily relies on food imports and faces extreme climatic conditions. Unlike many studies that broadly address renewable energy, this research specifically analyzes solar and wind energy, examining their distinct effects on various dimensions of food security, such as availability and stability. This approach enhances our understanding of whether renewable energy sources have a positive or a negative impact on food security. Additionally, the study explores both short-term and long-term effects of renewable energy shocks on food security, offering a dynamic perspective that can assist policymakers in developing adaptive and timely strategies.

The study is organized as follows: An introduction is presented in Section 1, followed by Section 2, which outlines the data, describes the variables, and details the methodological framework. Empirical results are presented in Section 3, and the study discussion, conclusions, and policy implications and limitations are detailed in Section 4, Section 5, and Section 6, respectively.

## 2. Methodology and Empirical Approach

We chose Saudi Arabia as the study area because the country faces significant challenges related to water scarcity, a key factor in food production. The country heavily relies on fossil-fueled water desalination for agriculture and drinking water.

### 2.1. Data

The annual time series data covering the period 2000–2022 is used. Food security data for pillars of food availability and stability, specifically focusing on the item average dietary energy supply adequacy (ADER), representing the food availability (in %), and cereal import dependency ratio % (CIDR), denoting the food stability measured in percent for a 3-year average (in %) were collected. Data used for this study were extracted from standardized food security sheets for Saudi Arabia from the FAO platform [43]. In addition, the energy data on the solar and wind energy measured in TWH growth-equivalent were collected from Our World in Data [44].

The study covers the period from 2000 to 2022, as official and standardized food security data, such as food security sheets, are consistently available only for these years. This period includes key global events affecting food security, such as the 2008 financial crisis, oil price fluctuations, and the COVID-19 pandemic, which have direct implications for food imports, production, and supply chains.

Analyzing food availability and stability can provide insights into the efficiency of existing agricultural policies, food distribution systems, and emergency response mechanisms in Saudi Arabia. The results obtained can help policymakers make informed decisions to enhance food security. Additionally, the study focuses on the link between food security and renewable energy consumption, as climate change presents significant challenges to food security by affecting crop yields, water availability, and agricultural practices. Investing in renewable energy can mitigate climate change by reducing greenhouse gas emissions, thereby supporting long-term food security.

Table 1 presents the summary statistics for the selected variables. Factual statistics are utilized to identify the presence of a normal distribution within the data sets analyzed of food security and renewable energy consumption.

Based on skewness, kurtosis, and Jarque–Bera test results for each variable, we observed that the negative skewness for ADER (−0.633) and CIDR (−0.110) indicate a slightly left-skewed distribution and opposite to solar and wind variables, and the positive kurtosis values for all variables suggests a distribution with weightier tails compared to a normal distribution (Table 1). Based on the Jarque–Bera test outcomes shown in the statistical table, the solar and wind variables do not have a normal distribution compared to the ADER and CIDR variables, which are more likely to follow a normal distribution (Table 1); accordingly, all variables are successfully transformed into natural logarithms.

**Figure 1:**
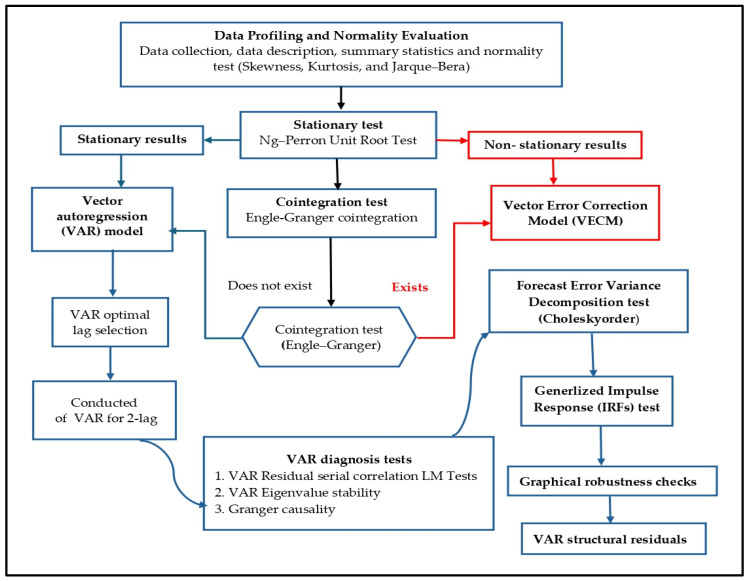
Structured outline of the research framework. Note: The red-highlighted test was not implemented in the analysis, because cointegration exists.

To analytically investigate the relationship between renewable energy consumption and food security in Saudi Arabia, a structured methodological framework was developed. Figure 1 illustrates the methodology, including the steps and models developed and adopted to conduct this study.

### 2.2. Econometrics Model

The model applied in this study is based on the Vector Autoregressive (VAR) model, which has proven to be a highly effective tool for analyzing interactions within a macroeconomic system since it is primarily disguised by [45]. As a primary step, before executing the VAR model, preliminary tests, such as unit root analysis, are conducted.

#### 2.2.1. Unit-Root Test

Investigating the stationarity of the studied variables is necessary for examining cointegration among the time series of the selected variables. We employed the Ng–Perron unit root test [46], as it is more powerful and better suited for small sample datasets [47]. We examine the properties of our Ng–Perron tests, which incorporate adjustments of four-unit root tests developed by Phillips [48] and Phillips & Perron [49], Phillips–Perron (PP) Zα and Zt, Bhargava R1, and Elliott–Rothenberg-Stock ERS, which is considered a feasible optimal point test, collectively referred to as the M tests. The tests are based on GLS detrended data $y_{t}^{d}$.

The formulas for testing these properties are:

(1) $$MZ_{\alpha }^{d}={\left(T^{-1}(y_{T}^{d}\right)}^{2}-c_{0})/2p$$

(2) $$MSB^{d}={\left(p/c_{0}\right)}^{\frac{1}{2}}$$

(3) $$MZ_{t}^{d}=MZ_{\alpha }^{d}\times MSB^{d}$$

(4) $$MPT_{T}^{d}={\bar{\left(v\right)}}^{2}p+\frac{\left((1-\bar{v)}T^{-1}\right){\left(y_{T}^{d}\right)}^{2}}{c_{0}}$$

Further, p (which relates to the number of lags used in the test, which helps adjust for autocorrelation in the residuals) and $c_{0}$ (a constant that represents the estimated coefficient in the model, related to the long-run variance) can take the formulas as:

(5) $$k=\sum_{t-2}^{T}\frac{{\left(y_{t-1}^{d}\right)}^{2}}{T^{2}}$$

(6) $$f_{0}=\sum_{j=-(T-1)}^{T-1}\emptyset \left(j\right)\cdot k\left(\frac{j}{l}\right)$$

where, $\bar{v}$ = −13.5, l is a smoothing parameter, functioning as a truncation lag in covariance weighting, and $\emptyset \left(j\right)$ is the jth auto-covariance of residuals. Two tests of Ng and Perron’s unit root are more powerful $MZ_{\alpha }^{d}$ and $MZ_{t}^{d}$.

#### 2.2.2. Basic VAR System

The study employs the advanced econometric vector autoregression (VAR) technique to assess the strength of the relationship between food security and renewable energy consumption and to analyze the causality of these variables in Saudi Arabia. The multivariate basic VAR approach is used after we confirm that the time series is stationary. The explanation for using the VAR model is that, by using a VAR model, we can examine the dynamics of food security and energy and how these variables influence each other over time. In addition, the VAR model is flexible and can capture short-term and long-term interactions between food security and renewable energy consumption. Both renewable energy consumption and food security have the potential to be endogenous. The VAR model can capture dynamic relationships between multiple variables, making it a versatile tool for analyzing complex systems. Additionally, basic VAR models can be applied to predict future values within the system and examine causality [50].

The basic VAR model is presented in the following reduced-simultaneous form:

(7) $${FE}_{t}=\alpha +A_{1}{FE}_{t-1}+\cdots +A_{\rho }{FE}_{t-\rho }+\omega_{t}=\alpha +A{FE}_{t-1}^{t-\rho }+\omega_{t}$$

where ${EF}_{t}$ the vector of endogenous factors, denoted as ADER, CIDR, SOLAR, and WIND, are predicted in this study. α is a constant term (M × 1), representing the interceptive vector. A is the coefficient matrix for the ith lag (M × n) in the backshift operator, with a lag length of p, and $\omega_{t}$ (n × 1) is the vector of white-noise error terms, i.e., consisting of reduced-form residuals that generally exhibit nonzero correlation. For a given VAR order ρ, estimation can be efficiently performed using equation-wise ordinary least squares (OLS), incorporating lag numbers based on lag length selection criteria from statistical information criteria, as shown in Equation (7).

After investigating the basic VAR, selecting the right lag length is crucial for determining over how many periods the model should be examined. We used standard criteria like the Akaike Information Criterion (AIC), Schwarz Bayesian Information Criterion (SBIC), Hannan–Quinn Information Criterion (HQIC), Likelihood Ratio criteria (LRC), and Final Prediction Error Criterion (FPE) to pick the best lag length.

### 2.3. Robust Analysis Checks

#### 2.3.1. Impulse Response Functions

We examine the forecast performance of VAR models emphasizing the impulse response functions (IRFs) and forecast-error variance decompositions (FEVDs) tests to investigate the dynamics of the VAR model for assessing the impact of variable shocks. The VAR analysis normally incorporates the estimation of IRFs and FEVDs which are the essential aspects of the VAR method. We follow the methods described in references [51,52] in establishing the IRFs and FEVDs for a 10-year forecast horizon (h).

The orthogonalized impulse response function is utilized to assess how the dependent variable reacts to shocks in each variable, specifically the impact of renewable energy consumption on food security. The impulse response at time horizon ‘h’ of the variables to an external shock to variable food security can be conveniently revealed by applying the Cholesky decomposition technique proposed by [45].

(8) $${FS}_{t}=\sum_{i=0}^{\infty }\emptyset_{i}\delta_{t-i}\left[\emptyset_{0}=I_{k}is\;the\;\left(K\times K\right)\;identity\;matrix\right]$$

whereas $\emptyset_{i}$

(9) $$\emptyset_{i}=\sum_{j=1}^{i}\emptyset_{i-j}A_{j}\left[i=1,2,3,\ldots \ldots \right]$$

where FS is food security variables (ADER or CIDR) $\emptyset_{i}$ are described as impulse responses of the model; Aj = 0 for j > ρ (for a k dimensional VAR (ρ) process); $\delta_{t}$ denotes the orthogonal residuals [53].

#### 2.3.2. Forecast-Error Variance Decompositions

The IRFs do not imply causation but clarify the probability of a shock on one variable affecting the other [54]. Recognition of operating the Cholesky decomposition of the covariance matrix is unique to ordering the variables in the VAR system [55]. Variance decomposition offers insight into the proportion of variations in the dependent variable that can be attributed to shocks. The variance decomposition analytical tool is instrumental in predicting the impact of exogenous shocks on the variables involved [56].

Within the VAR system, we use the h-step ahead predictor vector error calculation following [57], with its equation written as:

(10) $$d_{ij}(h_{t})=\frac{\delta_{jj}^{-1}\sum_{0}^{H-1}{\left(e_{it}^{\sim }A_{h}\sum^{e_{jt}}\right)}^{2}}{\sum_{0}^{H-1}{\left(e_{it}^{\sim }A_{h}\sum_{h}^{A}e_{it}^{\sim }\right)}^{2}}$$

where $d_{ij}(h_{t})$ is the h-step ahead forecast vector error designated at time t is influenced by i, j, and h (head); $\delta_{jj}^{-1}$ is a parameter of the variable associated with forecasting, $A_{h}$ is matrices related to the model. $e_{i}^{\sim }$ is error terms associated with the forecasting model. The orthogonalized shocks $e_{it}A_{h}$ (where A is a matrix) have a covariance matrix denoted as $I_{A}.$

## 3. Results

We begin by discussing the findings of the empirical investigation regarding the specified VAR model and its diagnosis in our study.

### 3.1. Unit Root Result

By comparing the test statistics of the unit root results for each variable with the critical values, the outcomes show that CIDR is stationary at both levels and first differences, ADER is stationary at the first difference, and WIND and SOLAR are stationary at the levels (Table 2).

The *p*-values for the Engle–Granger cointegration test shown in Table 3 are based on values reported in reference [58], assuming that the null hypothesis indicates no cointegration among the time series.

According to the results of Engle–Granger, considering the *p*-values of the variable LnWIND with the Tau-statistic (0.038) and Z-statistic (0.033) at a significance level of 5% in Table 3, there seems to be support for cointegration. This implies a long-term relationship between food security and wind renewable energy in Saudi Arabia. However, based on this analysis, LnADER, LnCIDR, and LnSOLAR do not show significant evidence of cointegration with the other variables in the study, suggesting a lack of long-term dynamics and interactions.

### 3.2. Lag-Order Selection Criteria

The optimal lag length is the lag length that produces the lowest FPE; therefore, lag 2 was chosen based on AIC, SIC, FPE, and LR standard criteria. It can be observed that both the Schwartz criteria and Akaike criteria provide the same results in both cases (Table 4). After the identification of the optimal lag length and integration properties has been established for the series, the VAR model can be applied; thus, the statistical tests of the VAR model are reliable, as seen in Table 5.

### 3.3. VAR Results

We used the VAR model to test the dynamic relationship between food security and renewable energy consumption. Table 5 presents the results of the VAR model with two optimal lags. The analysis of statistically significant coefficients confirmed by the Z-statistics reveals that LnADER(−1) shows a considerable positive impact on its own (Z-value = 7.468 at the 1% significance level). Conversely, LnADER−2 demonstrates a significant negative effect on its own (Z-value = −3.922 at the 1% significance level). Similarly, the LNCIDR(−1) has positive effects on its own at Z-values measured as 3.105 (at the 1% level).

Furthermore, LnSOLAR(−1) has a significant negative effect on LnADER (−2.157) and, in the same manner, has a significant positive effect on the LnCIDR (Z-value = 1.867 at a 5% significance level), and LnWIND (1.691) and its own (2.690). In addition, LnSOLAR(−2) has a significant positive effect on the LNCIDR (Z-value 1.682 at a 10% significance level). LnWIND(−2) has significant positive effects on the LnADER (Z value = 2.017 at a 5% significance level). These findings confirmed that one unit of renewable energy consumption from solar will significantly increase food stability by 36.5% in Saudi Arabia.

However, the analysis indicates that wind renewable energy affects only food availability; one unit of wind energy will increase food availability by 31.6%, and no statistically significant coefficients are associated with wind renewable energy consumption and food stability based on the Z-statistics provided.

We note that the root mean squared forecast errors (RMSFEs) are generally small across all models, except for the wind model. These RMSFEs are crucial for evaluating the overall accuracy of the model’s predictions. Additionally, the variables with significant coefficients and low standard errors are considered more reliable predictors to help evaluate the model’s goodness of fit and effectively explain the variability in endogenous variables.

### 3.4. VAR Diagnosis Analysis

In the context of VAR analysis, a VAR diagnosis analysis refers to investigations evaluating the adequacy and correctness of a VAR model that has been estimated using time series data. The study checks the residuals (errors) from the estimated VAR model for the serial correlation LM test. Table 6 shows that the results of Rao F-statistic and LRE statistics are not statistically significant and confirm that there is no serial correlation at lag 1 and 2 between observations that are h periods apart (*p*-values for LRE and Rao F-stat in lag 2 estimates as 0.403 and 0.4475; respectively for at lag h) or at various lagged time intervals within the range from lag 1 to lag h (*p*-values for LRE and Rao F-stat in lag 2 estimates as 0.552 and 0.373; respectively for at lag 1 to h). Therefore, the *p*-values are typically greater than 0.05 in both tests. This indicates that, in the absence of serial correlation, forecasting future values for the study variables may be relatively straightforward, since each observation is not dependent on past observations. Also, it is concluded that the VAR model is correctly specified and that the estimated coefficients are not biased due to autocorrelation.

**Figure 2:**
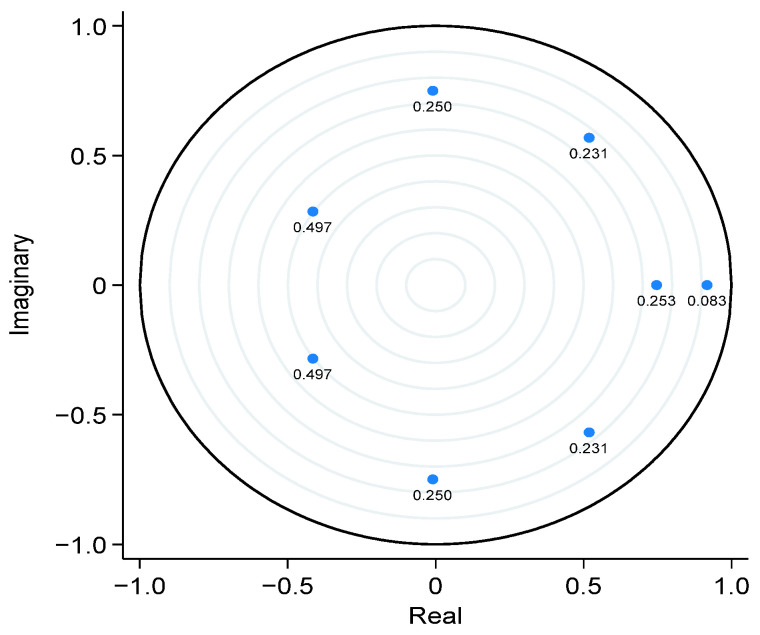
Roots of the companion matrix. Note: Points are labeled with their distances from the unit circle.

Moreover, the study estimated the stability of the VAR model and demonstrated that all eigenvalues (moduli) lie within the unit circle, exposing all modulus values as less than unity. This analysis confirms that the VAR system satisfies the essential stability criterion, ensuring stationarity and convergence properties (Table 7 and Figure 2).

### 3.5. Granger Causality Results

We employed Granger causality within a VAR context to explain potential causal links between the natural logarithms of renewable energy consumption and food security and their directions. This method helps identify significant variables within our model that exert a causal impact on others. Table 8 shows the findings from the asymmetric Granger causality analysis. The results reveal a significant causality running from LnCIDR to LnADER at the 10% significance level (ch^2^ = 150.1). The result also shows a significant bidirectional causality running from LnSolar to LnADER and vice versa (at a 1% level of significance). Nevertheless, the results indicated that the LnWIND does not Granger-cause the LnADER. In other words, there exist individuality conditions between these variables.

In addition, the LnADER, LnSolar, and LnWIND were not sensitive to the LnCIDR, i.e., do not Granger-cause the LnCIDR. This suggests that solar renewable energy has a Granger causality with food availability but not with food stability. In contrast, wind renewable energy does not exhibit Granger causality with either food availability or food stability. The inconsistent findings of the non-existence of causality can be attributed to the level of investment in renewable energy projects and agricultural development as well as access to funding sources, which can affect the outcomes in both sectors.

The findings suggest a possible assumption that an increase in solar renewable energy drives an increase in wind renewable energy, indicating a unidirectional relationship from LnSOLAR to LnWIND (ch^2^ = 6.6145 at 10% level of significance). The reason for these findings could be that solar and wind energy generation can be influenced by similar weather patterns. Also, solar and wind renewable energy are often considered complementary renewable energy sources in nature. In regions where both solar and wind resources are abundant, an increase in solar energy production may indicate favorable conditions for wind energy generation as well.

### 3.6. Forecast Error Variance Decomposition for Food Security and Renewable Energy Consumption

Table 9 presents the FEVDs for the selected variables LnADER, LnCIDR, LnSOLAR, and LnWIND over different forecasting periods. We suggest defining the term “short run” as 1–3 years and “long run” as 4–10 years. Based on the findings in Table 9, in the short run (1 year), food availability is entirely self-explanatory (100%). However, in the short run (3 years), variables such as food availability shock, food stability shock, renewable solar energy consumption shock, and wind energy consumption shock contributed approximately 61.909%, 16.568%, 21.246%, and 0.2767%, respectively, to food availability innovation.

In the long run (10 years), the initial contribution to food availability shock comes from internal factors. Food stability shock and renewable solar energy consumption shock contribute approximately 26.556% and 21.458%, respectively, to food availability. Simultaneously, wind renewable energy consumption contributed to food availability in the long run, accounting for a minimal percentage of 3.045%. This confirmed that investing in wind energy infrastructure may have long-term advantages for food availability by guaranteeing a stable and consistent energy supply for agricultural performance. This can contribute to enhanced efficiency in the food production sector.

In the short run, food availability contributed to the shock of food stability by half (50.452%), while the remaining half (49.548%) of the shock is attributed to food stability itself. This proves that, in the long run, food availability shock explains about 54.198% of food stability innovations, with slightly lower shocks of 5.596% and 3.511%, respectively, from solar and wind renewable consumption.

When examining solar energy FEVD, it becomes evident that short-run shocks in food availability account for 6.769% of the variations in solar energy, while the primary variations (93.196%) stem from the shocks of its variation. In the long run, food availability contributes 26.093% to solar innovation, whereas food stability contributes 14.215%. In the long run, the predominant share of solar FEVD is attributed to its shocks. Finally, the wind energy FEVD indicates that the short-run marginal contribution of food availability accounts for 0.893% of the variations in wind energy, and most variations (81.008%) arise from shocks to its own variation.

Food stability and solar renewable energy contributed about 11.239% and 6.661%, respectively, to the wind energy variation in the long run; food availability contributed 26.093% to solar innovation, whereas food stability contributed 14.215%. In the long run, food availability and stability shocks contribute to wind energy by 14.423% and 11.843%, respectively. Nearly half of the wind (49.349%) variation is attributable to its shocks.

Furthermore, as the forecasting periods progress (1–10 years), we observe a gradual shrinkage in the FEVD for LnADER. This implies that the uncertainty associated with LnADER becomes less important in interpreting the overall forecast error variance. In contrast, during succeeding periods, there is a gradual growth in the FEVD for LnCIDR, indicating that the uncertainty surrounding LnCIDR increases the importance of explaining the total forecast error variance. Similarly, the patterns for LnSOLAR and LnWIND change over the forecasting periods with varying FEVD values, reflecting the changing significance of these variables in describing the total forecast error variance. These insights are valuable in interpreting the relative importance of each variable in forecasting and decision-making processes in Saudi Arabia. Finally, we conclude that solar renewable energy consumption shocks have more persistent impacts in explaining the rapid increase in food security than wind renewable energy consumption shocks in both short and long runs.

**Figure 3:**
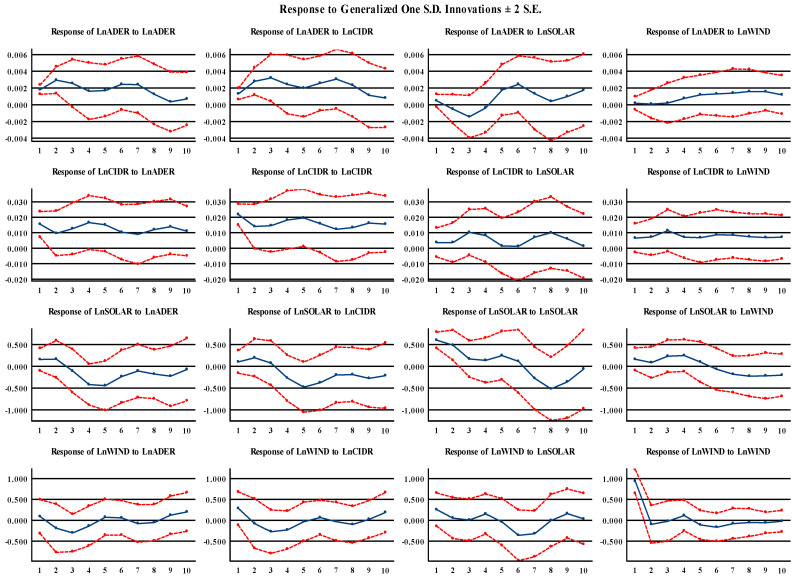
Impulse response functions. Note: The solid blue line symbolizes the impulse response of one variable (renewable energy consumption: solar and wind) to a one standard deviation shock to another variable and vice versa (food security: food availability and stability), whereas the red dashed lines signify standard error confidence intervals (CI) for the upper and lower limits of the 95% CI.

Finally, to enhance our understanding of the connection between food security and renewable energy consumption, we examined the impulse response function to verify how one variable responds to shocks created by other variables. Figure 3 illustrates the response to shocks originating among variables to generalize one SD innovation. We plot the dynamic impact of one standard deviation of renewable energy consumption shocks on Saudi Arabia’s food security over a horizon of 10 years, following several studies [59,60]. It seems that, as shown in Figure 2, food availability exhibits positive responses to a shock in solar renewable energy in the first horizon (year 1); this may be related to when solar energy is introduced or expanded, as farms may quickly benefit from lower energy costs, improved irrigation reliability, and reduced reliance on diesel pumps, boosting crop productivity and food availability in the short run. In relation to its negative responses in years 2–3, we suggest that, while solar-powered irrigation enhances access to water, it may unintentionally lead to the overexploitation of groundwater resources, lowering water tables, and threatening long-term agricultural sustainability. However, it displays a positive response in horizons 4–10 years, with the most significant response observed in the sixth horizon. Furthermore, food availability has shown a positive and steady increase in response to wind renewable energy. This result may be attributed to the increasing use of wind energy to power desalination plants, particularly in coastal areas where water scarcity limits agricultural productivity. In addition, wind energy projects typically involve larger-scale, centralized infrastructure which, once established, delivers consistent benefits with lower marginal costs.

Additionally, we observe that food stability responds positively to solar renewable energy shocks but negatively to wind renewable energy in the short and long run. This may be because food stability benefits more directly and immediately from solar energy due to its decentralized application and direct impact on agricultural water access. In contrast, wind energy’s more indirect role, combined with geographic and infrastructure mismatches, may explain its persistent negative effect on food stability over time.

### 3.7. Graphical Robustness Checks

**Figure 4:**
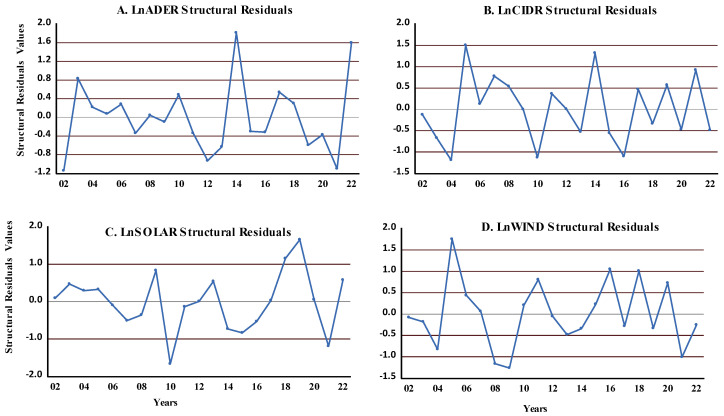
VAR structural residuals. Note: All figures (**A**–**D**) alternate between positive and negative values, it implies that the variable is fluctuating between growth and decline, positive and negative trends, or periods of improvement and deterioration.

VAR structural residuals are crucial for conducting robustness checks to ensure the model is reliable and accurately captures the links with the variables in the data. Monitoring the behavior of structural residuals over time can help evaluate the stability of the VAR model. We observed sudden changes in the residuals of food security and renewable energy (Figure 4), which confirmed the VAR model’s stability (Figure 2).

Considering food security, availability, and/or stability, rapid transitions in residuals could suggest unexpected economic shocks impacting food security, such as sudden changes in prices, food availability, or income levels. Also, seasonal variations in food production or consumption patterns could contribute to sudden changes in residuals from positive to negative values, to food security. Analyzing these changes can help understand the underlying dynamics affecting food security and identify areas requiring attention or intervention.

## 4. Discussion

The intersection of energy and food security is increasingly critical in today’s global landscape, particularly in regions facing severe climatic challenges and with heavy reliance on food imports. This study sheds light on the complex dynamics between renewable energy sources, specifically solar and wind, and their impact on food security dimensions such as availability and stability.

In our study, we found that solar energy significantly enhances food availability, aligning with findings reported in [23], and that consuming renewable energy sources positively impacts food security in the short run; however, this effect does not persist in the long run. This discrepancy can be resolved by considering appropriate factors. In the short term, solar energy reduces operational costs, improves irrigation, and supports crop productivity. Over time, however, challenges such as system degradation, lack of maintenance infrastructure, limited technical capacity, and policy uncertainty can diminish these benefits. Therefore, while solar energy holds strong potential to improve food availability, its long-term impact depends on sustained investments in technology upkeep, local capacity-building, and supportive regulatory frameworks. These results are supported by [61] who found that solar energy consumption is significantly associated with increased crop production, higher farmer income, and improved food security.

In comparison with our results, ref. [62] argued that expanding solar infrastructure may reduce land for food production, deepen import reliance, and negatively impact food security in GCC countries.

However, our analysis uniquely highlights the impacts of solar and wind energy, which are factors often ignored in previous studies. This difference is crucial, especially in the context of regions that experience varying climatic conditions. Furthermore, while [23] suggested that renewable energy principally benefits food security in the short run but not in the long run, our findings indicate that long- and short-term impacts are also significant, highlighting the need for flexible policy strategies.

Another study focused on wind renewable energy and agreed with our results, revealing that one unit of wind energy consumption could increase food availability by 31.6%. However, it found no statistically significant relationship between wind energy consumption and food stability, suggesting that while food availability may improve, other factors could influence food stability [63]. Although wind energy contributes to increasing food availability, it does not have a significant impact on food stability.

GCC countries in general are considered as arid, oil-dependent economies with a desert climate; the GCC region receives high solar irradiation, with average daily radiation exceeding 6 kWh/m^2^ and 80–90% clear weather conditions throughout the year, making solar energy a preferred choice. However, wind energy faces challenges such as lower wind speeds in some areas and limited infrastructure for large-scale deployment [64]. A study performed by [64] in Oman showed that solar energy has been the only source of renewable energy production levels in Oman since 2017 and has a significant indirect effect on food security.

In contrast, our causality findings indicated a bidirectional Granger causality, particularly between renewable energy equity and agricultural commodities across different frequencies, highlighting the crucial link between renewable energy investment and food security dynamics. Research has shown that there is a bidirectional relationship between renewable energy consumption and agricultural output, suggesting that investments in renewable energy can enhance agricultural productivity, which in turn can influence energy consumption patterns and food security [65]. This supports our findings of a crucial link between renewable energy and food security.

Our findings indicate that renewable solar energy consumption shocks account for approximately 21.458% of the variation in food availability over the long run. This result highlights the substantial role that solar energy plays in influencing agricultural outcomes, particularly in the context of food security and sustainable development. These findings are consistent with the results reported by [66], which demonstrated that renewable energy shocks, particularly from sources like solar and bioenergy, can explain between 25% and 30% of the variability in long-term food production growth. The slight difference in magnitude (21.458% vs. 25–30%) could be attributed to geographic and methodological differences and estimation techniques.

Increasing water use for services in Saudi Arabia increases food [20], and the rising threat of food insecurity remains a key challenge for policymakers [67]. However, Mujtaba et al. [68] identified policy, regulatory, technical, and financial barriers as the primary constraints delaying the adoption of renewable energy in the agri-food processing sector, thereby limiting its potential to enhance food security.

## 5. Conclusions

The rising global demand for energy and food has led to increasing attention to the nexus between renewable energy consumption and food security. This study examines the effects and shocks of renewable energy, particularly solar and wind energy, on Saudi Arabia’s food security by examining the short-term and long-term effects on food availability and stability. The annual time series data for the period 2000–2022 was utilized. The model applied in this study is based on the Vector Autoregressive (VAR) model and its environment.

The Ng–Perron unit root results confirm that CIDR, WIND, and SOLAR are stationary at the levels while the ADER is stationary at the first difference, which proves the adoption of the VAR analysis with the optimal 2 lag length. Engle–Granger cointegration results indicate a long-term relationship between food security and wind renewable energy consumption in Saudi Arabia. The VAR results indicate that wind renewable energy affects only food availability, and no statistically significant coefficients are associated with wind renewable energy consumption and food stability. This might be because, in Saudi Arabia, solar and wind renewable energy initiatives are typically geared towards electricity generation and industrial applications rather than agriculture.

The Granger causality results indicated that solar renewable energy has a Granger causality with food availability but not with food stability. FEVD results indicate that long-run food availability shocks are primarily driven by internal factors. In the short run, food availability contributes to food stability shocks. Over time, the forecast error variance for LnADER gradually decreases. Also, we concluded that solar renewable energy consumption shocks have more persistent impacts in explaining the rapid increase in food security than wind renewable energy consumption shocks in both short and long runs. Food availability shows a positive and steady response to wind energy, while food stability responds positively to solar energy but negatively to wind energy in both the short and long term. The stability of the VAR model suggests that sudden changes in the residuals of food security and renewable energy may indicate unexpected economic shocks affecting food prices, availability, or income levels.

Saudi Arabia’s NREP can enhance food stability by integrating technologies of solar energy for irrigation and wind energy for desalination. These technologies offer sustainable water solutions for agriculture, especially in agriculture-rich regions and coastal areas, helping reduce reliance on fossil fuels and groundwater. Additionally, this integration can be achieved through collaboration with stakeholders, supported by well-planned and informative strategies. Consequently, this approach promotes more resilient and sustainable food systems aligned with the goals of Vision 2030.

## 6. Policy Implications and Limitations

This research reveals how renewable energy development influences food security outcomes, offering actionable insights for policymakers. The findings can guide integrated policies that optimize land use, energy investments, and food supply chain resilience. In addition, future research should explore emerging technologies in wind and solar energy that can enhance efficiency and sustainability while minimizing adverse effects on food security. Furthermore, although the NREP primarily targets energy, its connection to the food–energy nexus positions it as a crucial asset for improving food stability in Saudi Arabia. The NREP can address significant gaps in the Kingdom’s food security framework by integrating and promoting sustainable, climate-smart, and locally driven agricultural practices. This integration can be achieved through collaboration with stakeholders, effective planning, and the implementation of informed strategies. Such efforts would strengthen the Kingdom’s resilience to food supply disruptions. Future policy efforts should also explore advanced renewable technologies that optimize land use, boost efficiency, and ensure long-term food system sustainability in arid and resource-constrained settings.

The study faces crucial limitations that affect the real findings. The data available for solar and wind energy are limited, leading to difficulties in establishing clear relationships with food security. The feasibility of integrating renewable energy with food security depends on existing infrastructure and technological advancements, which may not be uniformly available. By addressing these limitations and implementing the recommendations, the study can provide a more robust and practical framework for understanding the interconnections between renewable energy and food security.

Similarly, one significant constraint is our reliance on aggregate energy data, which may conceal variations at the micro level. These limitations can affect our findings’ robustness and applicability across different sectors. The use of aggregated energy data may lead to bias by hiding regional and sectoral differences. For example, the impacts of renewable energy on food security may differ between rural and urban areas, leading to differences that aggregate data cannot capture. This limitation may cause the findings to ignore significant details, such as the specific advantages of decentralized renewable energy techniques for smallholder farmers, which can reduce the accuracy and effectiveness of policy suggestions. To enhance the understanding of the food security–renewable energy nexus, we recommend that future investigations focus on micro-level studies that explore specific sectors in greater detail. Such analyses could yield richer insights and inform more targeted policy recommendations.

## Figures and Tables

**Table 1:** Summary statistics of the selected variables.

Statistic Term	ADER	CIDR	SOLAR	WIND
Mean	2380.478	86.870	2.061	2.706
Median	2398.000	88.000	0.332	0.752
Maximum	2424.000	103.600	10.807	11.768
Minimum	2312.000	69.900	−0.571	−0.650
Std. Dev.	37.822	10.430	3.018	3.603
Skewness	−0.633	−0.111	1.413	1.369
Kurtosis	1.910	1.888	4.205	3.626
Jarque–Bera	2.675	1.233	9.044	7.558
Probability	0.263	0.540	0.011	0.023
Sum	54,751.000	1998.000	47.402	62.236
Sum Sq. Dev.	31,471.740	2393.249	200.334	285.553
Observations	23	23	23	23

**Table 2:** Ng-Perron results.

Ng–Perron Test Statistics (Intercept Model) at a Level					Ng–Perron Test Statistics (Intercept Model) at First Difference			
Test Variable	MZa	MZt	MSB	MPT	MZa	MZt	MSB	MPT
LnADER	−2.742	−0.913	0.333	8.059	−55.705	−4.988	0.090	1.105
LnCIDR	−13.717	−2.481	0.181	2.288	−16.713	−2.890	0.173	1.470
LnSOLAR	−4.990	−1.448	0.290	5.194	−11.945	−2.197	0.184	2.939
LnWIND	−10.801	−2.302	0.213	2.350	−7.948	−1.962	0.247	3.194

Note: At 1%, asymptotic critical values = −13.800, −2.580, 0.174, and 1.780 for MZa, MZt, MSB, and MPT; respectively. At 5%, asymptotic critical values = −8.100, −1.980, 0.233, and 3.170 for MZa, MZt, MSB, and MPT; respectively. At 10% asymptotic critical values = −5.700, −1.620, 0.275, and 4.450 for MZa, MZt, MSB, and MPT; respectively. Source: Authors’ calculations (2025).

**Table 3:** Engle–Granger cointegration test results.

Endogenous Variables	Tau-Statistic	^a^ *p*-Values	Z-Statistic	*p*-Values
LnADER	−4.493	0.063	−17.99193	0.1599
LnCIDR	−3.397	0.304	−13.56217	0.4227
LnSOLAR	−2.701	0.603	−11.29017	0.5981
LnWIND	−4.801	0.038 ***	−22.93033	0.033 ***
**Intermediate Results**				
Tests	LnADER	LnCIDR	LnSOLAR	LnWIND
^b^ Rho (ρ) (rho) − 1	−0.818	−0.616	−0.5132	−1.0423
^c^ Rho (ρ) S.E.	0.182	0.1814	0.1899	0.217
Residual variance	8.82 × 10^−6^	0.0006	0.5198	0.705
Long-run residual variance	8.82 × 10^−6^	0.0006	0.5198	0.705
** Number of stochastic trends	4	4	4	4

Note: ^a^ MacKinnon *p*-values [58] supposing the null hypothesis indicates series are not cointegrated, *** significance at level 5%, ** The number of stochastic trends in the asymptotic distribution. ^b^ Rho (ρ) represents the coefficient of the lagged difference term in the cointegration equation. ^c^ Rho (ρ) S.E. is the standard error of the coefficient. Source: Authors’ calculations (2025).

**Table 4:** Optimal lag selection criteria for food security and renewable energy consumption.

Estimation Lag Order Statistics								
Lag	LogL	LR	FPE	AIC	HQIC	SBIC	df	*p*-Value
0	7.669		9.2e	8.00	−4.852	−4.825		
1	118.367	141.4	2.2 × 10^−10^	−10.930	−10.793	−9.940	16	0.00
2	153.247	69.759 *	3.8 × 10^−11^ *	−13.027 *	−12.782 *	−11.247 *	16	0.00

Note: Endogenous: LnCIDR LnADER LnSolar LnWind. Exogenous variables: Constant. * Indicates lag order selected by the LR, FPE, AIC, SC, and HQ criterion. Source: Authors’ calculations (2025).

**Table 5:** VAR model for renewable energy consumption and food security.

Independent Variables (Exogenous)	Dependent Variables (Endogenous)			
	LnADER	LnCIDR	LnSOLAR	LnWIND
LnADER(−1)	1.054 (0.141) [7.468] ***	1.756 (0.317) [5.549] ***	−2.183 (0.302) [−7.239] ***	0.036 (0.183) [0.198]
LnADER(−2)	−0.521 (0.133) [−3.922] ***	0.291 (0.341) [0.852]	−0.373 (0.516) [−0.723]	0.484 (0.207) [2.341]
LnCIDR(−1)	0.042 (0.109) [0.388]	0.759 (0.244) [3.105] ***	0.026 (0.233) [0.110]	−0.057 (0.141) [−0.404]
LnCIDR(−2)	0.005 (0.103) [0.044]	0.051 (0.263) [0.193]	0.206 (0.399) [0.517]	−0.108 (0.160) [−0.679]
LnSOLAR(−1)	−0.194 (0.090) [−2.157] **	0.376 (0.201) [1.867] **	0.516 (0.192) [2.690] **	0.196 (0.116) [1.691] *
LnSOLAR(−2)	0.069 (0.084) [0.817]	0.365 (0.217) [1.682] *	−1.285 (0.328) [−3.914] ***	0.238 (0.131) [1.812]
LnWIND(−1)	−0.237 (0.166) [−1.424]	0.184 (0.373) [0.493]	−0.044 (0.355) [−0.125]	−0.143 (0.215) [−0.665]
LnWIND(−2)	0.316 (0.157) [2.017] **	−0.394 (0.402) [−0.980]	0.002 (0.609) [0.004]	0.509 (0.244) [2.090] **
Constant	941.480 (184.138) [5.113] ***	−93.074 (142.190) [−0.655]	236.151 (117.098) [2.017] **	−166.250 (217.048) [−0.766]
RMSFE	0.000813	0.025469	0.426824	1.21787
R-squared	0.996	0.9730	0.9864	0.760
Chi^2^	11,667.92 ***	647.7489 ***	1303.26	57.09254
Log-likelihood	153.2501			
FPE	3.83 × 10^−11^			
Det (Sigma_ml)	4.73 × 10^−13^			
AIC	−24.82375			
HQIC	−24.60549			
SBIC	−23.24086			

Note: The test statistic: (Std. err values) is in parentheses and [Z values] in square brackets. RMSFE: It denotes the forecast errors estimated by the difference between the predicted and actual values, which are relatively small compared to the scale of the data. ***, **, and * represent significance levels at 1%, 5%, and 10%; respectively. Source: Authors’ calculations (2025).

**Table 6:** VAR residual serial correlation LM tests.

Null hypothesis: No serial correlation at lag h						
Lag	LRE * stat	df	*p*-value	Rao F-stat	df	*p*-value
1	11.99049	16	0.7446	0.683857	(16, 15.9)	0.7720
2	16.73130	16	0.4032	1.069668	(16, 15.9)	0.4475
Null hypothesis: No serial correlation at lags 1 to h						
Lag	LRE * stat	df	*p*-value	Rao F-stat	df	Prob.
1	11.99049	16	0.7446	0.683857	(16, 15.9)	0.7720
2	45.69779	32	0.0552	1.405993	(32, 5.3)	0.3734

* Edgeworth expansion corrected likelihood ratio statistic is the likelihood ratio statistic corrected using the Edgeworth expansion. Source: Authors’ calculations (2025).

**Table 7:** Eigenvalue stability condition of the VAR model.

Root	Modulus
0.225244 − 0.939224i	0.965855
0.225244 + 0.939224i	0.965855
0.961982	0.961982
0.781949 − 0.448352i	0.901368
0.781949 + 0.448352i	0.901368
−0.169970 − 0.528043i	0.554724
−0.169970 + 0.528043i	0.554724
−0.138230	0.138230

Note: The eigenvalues consist of real and imaginary components, with the left values representing the real parts and the right values indicating the imaginary parts, including their respective signs. All eigenvalues fall within the unit circle, confirming that the VAR model satisfies the stability criteria, as no root lies outside the unit circle. Source: Authors’ calculations (2025).

**Table 8:** Granger causality relationships between food security and renewable energy.

	Excluded	chi^2^	Prob > chi^2^	The Results of Causality Run	Direction
LnADER	LnCIDR	150.100	0.00 ***	CIDR → ADER	Unidirectional
	LnSOLAR	52.770	0.00 ***	Solar ←→ ADER	Bidirectional
	LnWIND	0.848	0.65	No causality	Individuality
	ALL	319.330	0.00 ***	REC ←→ ADER	Bidirectional
LnCIDR	LnADER	0.893	0.64	No causality	Individuality
	LnSOLAR	1.388	0.50	No causality	Individuality
	LnWIND	0.9188	0.63	No causality	Individuality
	ALL	4.763	0.58	No causality	Individuality
LnSolar	LnADER	18.457	0.00 ***	ADER ←→ Solar	Bidirectional
	LnCIDR	0.600	0.74	No causality	Individuality
	LnWIND	1.499	0.47	No causality	Individuality
	ALL	65.480	0.00 ***	FS ←→ Solar	Bidirectional
Lnwind	LnADER	2.307	0.32	No causality	Individuality
	LnCIDR	2.337	0.31	No causality	Individuality
	LnSOLAR	6.615	0.04 *	Solar → Wind	Unidirectional
	ALL	27.962	0.00 ***	FS ←→ Wind	Bidirectional

Note: Null hypothesis of non-Granger-causality through the Wald test. *** and * represent significant levels at 1%, and 10%, respectively. Source: Authors’ calculations (2025).

**Table 9:** FEVD results.

Period	FEVD for LnADER				FEVD for LnCIDR			
	LnADER	LnCIDR	LnSOLAR	LnWIND	LnADER	LnCIDR	LnSOLAR	LnWIND
1	100.000	0.000	0.000	0.000	50.452	49.548	0.000	0.000
2	80.955	6.919	11.768	0.358	48.622	49.942	0.299	1.137
3	61.909	16.568	21.246	0.277	49.341	40.390	5.612	4.657
4	57.996	22.503	19.120	0.381	55.637	35.422	5.394	3.547
5	56.378	22.147	20.905	0.570	56.110	36.449	4.479	2.962
6	56.010	20.173	23.302	0.515	53.836	38.703	3.957	3.503
7	56.127	22.750	20.469	0.655	52.867	38.456	4.785	3.892
8	53.067	26.817	18.581	1.535	53.395	36.439	6.446	3.720
9	50.742	27.647	18.933	2.678	54.790	35.678	6.044	3.488
10	48.941	26.556	21.458	3.045	54.199	36.693	5.597	3.511
**Period**	**FEVD for LnSOLAR**				**FEVD for LnWIND**			
1	6.769	0.035	93.196	0.000	0.893	11.233	6.866	81.008
2	8.300	2.029	88.208	1.463	4.678	11.029	7.514	76.779
3	8.563	7.877	80.000	3.560	12.919	10.577	7.502	69.002
4	23.156	5.771	63.470	7.603	13.189	12.774	9.791	64.247
5	30.287	7.870	55.173	6.670	13.326	13.708	9.895	63.071
6	30.365	12.627	51.056	5.952	11.972	12.168	19.690	56.170
7	29.070	13.498	51.711	5.721	11.529	11.371	24.942	52.157
8	26.599	11.997	56.333	5.071	11.648	11.806	24.751	51.794
9	26.746	12.258	56.142	4.855	12.275	11.946	25.133	50.646
10	26.093	14.215	54.373	5.318	14.423	11.844	24.384	49.349

Note: Cholesky Ordering: LnADER LnCIDR LnSOLAR LnWIND. Source: Authors’ calculations (2025).

## Data Availability

The datasets used and analyzed in the current study were derived from the public domain open access resources’ repository FAOSTAT free online from: https://www.fao.org/faostat/en/#data, accessed on 24 January 2025, and Our World in Data at: https://ourworldindata.org/energy, accessed on 24 January 2025.
